# Emotional Reactivity and Emotion Regulation in Relation to Health-Related Quality of Life After Burn Injury: A Cross-Sectional Observational Study

**DOI:** 10.3390/ebj7040051

**Published:** 2026-09-29

**Authors:** Nguyen Tien Dung, Pham Thi Mai Phuong, Nguyen Thanh Chung, Hoang Duc Minh

**Affiliations:** Vietnam National Burn Hospital, Hanoi 10000, Vietnam; ntzung_0350@yahoo.com (N.T.D.);

**Keywords:** burn injury, health-related quality of life, emotional reactivity, emotion regulation, TBSA, BSHS-B, EQ-5D-5L, rehabilitation

## Abstract

**Highlights:**

**What are the main findings?**

- Greater TBSA was associated with higher emotional reactivity, whereas burn depth was not significantly associated with emotional reactivity.
- Higher emotional reactivity was consistently associated with poorer burn-specific and generic HRQoL, while psychosocial characteristics provided additional explanatory value beyond demographic and clinical measures.

**What are the implications of the main findings?**

- Psychosocial functioning should be considered alongside conventional measures of burn severity when assessing HRQoL after burn injury.
- Integrating assessment of emotional reactivity and emotion-regulation strategies into multidisciplinary burn rehabilitation may help identify patients with greater psychosocial needs and support more individualized care.

**Abstract:**

**Introduction:** Burn severity alone may not explain differences in health-related quality of life (HRQoL) after burn injury. We examined whether emotional reactivity and emotion-regulation strategies were associated with HRQoL beyond burn severity. **Methods**: This cross-sectional study included 256 adults with burn injury treated at the Vietnam National Burn Hospital (2024–2025). Total body surface area (TBSA) and burn depth represented severity. Emotional reactivity and emotion-regulation strategies were assessed using the Emotion Reactivity Scale (ERS) and Emotion Regulation Questionnaire-Short Form. Burn-specific and generic HRQoL were assessed using the Burn-Specific Health Scale–Brief (BSHS-B) and EuroQol five-dimensional five-level (EQ-5D-5L). **Results**: TBSA was positively associated with ERS (r = 0.30, *p* < 0.001), whereas burn depth was not (*p* = 0.18). Higher ERS was associated with poorer BSHS-B Function (r = −0.34), Affect and Relations (r = −0.39), Skin Involvement (r = −0.28), and EQ-5D-5L (r = −0.32; all *p* < 0.001). Cognitive reappraisal showed positive, whereas expressive suppression showed negative associations with HRQoL. In hierarchical regression for BSHS-B Affect and Relations, psychosocial variables increased adjusted R^2^ from 0.168 to 0.291 (Δadjusted R^2^ = 0.123, *p* < 0.001). **Conclusions:** Emotional reactivity and emotion-regulation strategies were associated with HRQoL and provided additional explanatory information beyond burn severity, supporting their consideration in individualized rehabilitation.

## 1. Introduction

Burn injury is a major traumatic condition with consequences extending beyond acute tissue damage and survival. As survival has improved, the goals of burn care have increasingly expanded toward functional recovery, psychosocial adjustment, and health-related quality of life (HRQoL) [1,2]. Psychosocial adjustment after burn injury encompasses multiple domains, including psychological distress, quality of life, interpersonal functioning, return to work, and adaptation to altered appearance and physical limitations [1]. Consequently, psychosocial assessment and appropriate psychological support are increasingly recognized as important components of comprehensive burn care [3].

HRQoL after burn injury is determined by both injury-related and psychological factors. A systematic review of 32 studies found that burn severity, post-burn depression, post-traumatic stress symptoms, coping, emotional and social support, neuroticism, and employment were associated with HRQoL, indicating that physical injury severity and psychological responses provide complementary information about recovery [2]. However, most previous research has focused on established psychological conditions or broad psychosocial outcomes rather than on more specific dimensions of emotional functioning.

Emotional reactivity may represent one such dimension. It refers to individual differences in the sensitivity, intensity, and persistence of emotional responses and can be assessed using the Emotion Reactivity Scale (ERS), a validated 21-item measure with demonstrated internal consistency and construct-related validity [4]. In the context of burn injury, heightened emotional reactivity may be particularly relevant because patients must adapt to pain, functional impairment, altered appearance, uncertainty about recovery, and disruption of interpersonal and social roles. Nevertheless, the role of emotional reactivity in relation to burn severity and HRQoL has received limited attention.

Emotion regulation provides a complementary perspective on psychosocial adaptation. Cognitive reappraisal and expressive suppression represent distinct strategies for regulating emotional responses and are assessed by the Emotion Regulation Questionnaire–Short Form (ERQ-S) [5]. Although emotion-regulation strategies have been extensively studied in psychological research, their relationships with emotional reactivity and multidimensional HRQoL following burn injury remain insufficiently characterized. In particular, it remains unclear whether these psychosocial characteristics contribute additional explanatory information beyond conventional indicators of burn severity, such as total body surface area (TBSA) and burn depth.

This gap is clinically relevant because patients with comparable anatomical injury may experience substantially different psychosocial outcomes. Routine psychosocial screening has been advocated in burn care because psychological needs may be substantial and are not necessarily reflected by clinical severity alone [3]. Recent evidence from low- and middle-income countries further emphasizes the substantial HRQoL burden among burn survivors and the importance of rehabilitation in settings where access to comprehensive psychosocial services may be limited [6].

The conceptual framework of this study was informed by established biopsychosocial and health-related quality-of-life models. In burn populations, the biopsychosocial framework emphasizes the interplay between injury-related characteristics, psychological functioning, coping, and subsequent adjustment [7]. The Wilson–Cleary model further provides a framework linking clinical and individual-level factors with patient-reported health-related quality of life [8]. Consistent with these frameworks and previous evidence that both burn severity and psychological factors are associated with HRQoL after burn injury [2], we conceptualized burn severity and psychosocial characteristics as complementary domains contributing to HRQoL. Accordingly, the present study aimed to examine the associations of clinical burn severity, emotional reactivity, and emotion-regulation strategies with HRQoL within an integrated biopsychosocial framework. Specifically, we sought to determine whether emotional reactivity and emotion-regulation strategies were associated with HRQoL after adjustment for relevant demographic and clinical characteristics, and whether these psychosocial variables provided additional explanatory value beyond conventional measures of burn severity.

## 2. Materials and Methods

### 2.1. Study Design

This study was designed as a cross-sectional observational study of adult patients with burn injury treated at the Vietnam National Burn Hospital, Hanoi, Vietnam. The study was guided by biopsychosocial and health-related quality-of-life frameworks [7,8] and examined the relationships among clinical indicators of burn severity, represented by TBSA and burn depth, psychosocial characteristics, including emotional reactivity and emotion-regulation strategies and HRQoL.

The primary analysis evaluated the associations of emotional reactivity and emotion-regulation strategies with HRQoL after adjustment for relevant demographic and clinical characteristics. A secondary analysis assessed whether psychosocial variables provided additional explanatory value beyond conventional measures of burn severity. Given the observational nature of the analysis, all associations were interpreted as non-causal. The conceptual and analytical framework of the study is presented in Figure 1.

### 2.2. Data Collection

Clinical, psychosocial and HRQoL data were collected using a standardized data-collection protocol. Clinical characteristics were extracted from medical records, while psychosocial and HRQoL information was obtained through structured patient-reported assessments. Psychosocial and HRQoL assessments were conducted during hospitalization after patients were clinically stable and able to complete the questionnaires. The timing of assessment was not fixed to a specific number of days after burn injury or hospital admission but was determined by the individual clinical course and ability to participate. Participants completed the questionnaires independently or received standardized assistance when necessary.

The study-specific data-collection form was developed by the research team based on the study objectives and relevant validated instruments. The preliminary version was reviewed by a multidisciplinary expert panel for content relevance, clarity, clinical appropriateness, and cultural comprehensibility, followed by pilot testing in 10 eligible burn patients to assess comprehensibility, acceptability, and feasibility. Feedback from both the expert review and pilot assessment was incorporated to refine the wording, sequence, and instructions before finalization and formal data collection.

The finalized assessment incorporated the ERS and the ERQ-S Form as standardized measures of emotional reactivity and emotion-regulation strategies, respectively. The ERS assesses emotional sensitivity, intensity, and persistence, whereas the ERQ-S evaluates cognitive reappraisal and expressive suppression.

### 2.3. Participants

The analytical cohort consisted of 256 adult patients with burn injury treated at the Vietnam National Burn Hospital, Hanoi, Vietnam, for whom clinical, psychosocial and HRQoL data were available for the present study. Clinical, psychosocial and HRQoL data were collected from October 2024 to January 2025 from hospitalized burn patients aged 18–80 years treated at the Adult Burn Department and the Department of Rehabilitation.

Patients were eligible if they were aged 18–80 years, had a clinically confirmed burn injury with documented TBSA and burn depth, were sufficiently clinically stable and cognitively able to complete the psychosocial assessment, were able to provide reliable self-reported information, and had sufficient data for the primary analysis.

Patients were excluded if they were unable to complete the psychosocial assessment because of impaired consciousness or severe clinical instability, were unable to provide reliable self-reported information, declined participation, or had insufficient information for the primary analysis.

Sample size consideration: The sample size was determined by the number of eligible patients available during the predefined study period. All eligible patients meeting the prespecified inclusion and exclusion criteria were included in the cross-sectional analysis. This census-based approach was used to maximize the available sample and statistical information. No formal prospective sample size calculation was performed.

### 2.4. Clinical and Psychosocial Assessment

Emotional reactivity was assessed using the ERS, a 21-item self-report instrument comprising three dimensions: emotional sensitivity, emotional intensity, and emotional persistence [4]. Each item is rated on a 5-point scale from 0 (“not at all like me”) to 4 (“completely like me”), yielding a total score ranging from 0 to 84. Higher scores indicate greater emotional reactivity. The total ERS score was used as the primary measure, while its component dimensions were examined in secondary analyses.

Emotion-regulation strategies were assessed using the ERQ-S, a six-item instrument comprising two dimensions: cognitive reappraisal and expressive suppression [5]. Each item is rated on a 7-point Likert scale from 1 (“strongly disagree”) to 7 (“strongly agree”). Each subscale comprises three items and therefore ranges from 3 to 21, with higher scores indicating greater use of the corresponding regulatory strategy. In the present sample, internal consistency was acceptable for the ERS (Cronbach’s α = 0.94), cognitive reappraisal (α = 0.76), and expressive suppression (α = 0.72).

### 2.5. Health-Related Quality of Life

HRQoL was assessed using the Burn-Specific Health Scale–Brief (BSHS-B) as the primary burn-specific outcome and the EuroQol five-dimensional five-level questionnaire (EQ-5D-5L) as a secondary generic measure [9,10]. BSHS-B was used to assess multidimensional burn-related functioning and psychosocial well-being, with the Function, Affect and Relations, and Skin Involvement domains included in the present analysis [9]. Higher BSHS-B scores indicate better burn-specific HRQoL. The EQ-5D-5L assesses mobility, self-care, usual activities, pain/discomfort, and anxiety/depression [10]. The Vietnamese value set was used to derive the EQ-5D-5L health-state index [11].

### 2.6. Statistical Analysis

Statistical analyses were performed using R Statistical Software, version 4.5.1 (R Foundation for Statistical Computing, Vienna, Austria). Continuous variables were summarized as mean ± standard deviation (SD) or median with interquartile range (IQR), as appropriate, while categorical variables were presented as frequencies and percentages.

The relationships among burn severity, emotional reactivity, emotion-regulation strategies, and HRQoL were initially examined using Pearson correlation coefficients, according to the distribution and measurement characteristics of the variables. For associations involving the dichotomous burn-depth variable and continuous measures, the point-biserial correlation coefficient was used. Differences in psychosocial measures across clinically relevant categories of burn severity were assessed using appropriate parametric or non-parametric group-comparison tests.

The analytical framework included TBSA and burn depth as conventional indicators of burn severity. The primary multivariable analysis used hierarchical multivariable linear regression to further examine the associations of emotional reactivity and emotion-regulation strategies with the BSHS-B Affect and Relations domain, selected because of its direct relevance to the psychosocial constructs examined in this study. Other HRQoL outcomes were examined using correlation analyses as complementary outcomes. Three sequential models were specified for the primary analysis. Model 1 included age and sex; Model 2 additionally incorporated TBSA and burn depth; and Model 3 further included emotional reactivity, cognitive reappraisal, and expressive suppression.

Standardized regression coefficients (β) were reported for each model, with 95% confidence intervals (CIs) and *p*-values presented for the final model, together with adjusted R^2^. The incremental explanatory contribution of psychosocial variables was assessed by comparing changes in adjusted R^2^ across the sequential models.

The assumptions of linear regression, including linearity, homoscedasticity, normality of residuals, and multicollinearity, were assessed before interpretation. Multicollinearity was evaluated using the variance inflation factor (VIF), with VIF < 5 considered acceptable and VIF ≥ 5 indicating potentially problematic multicollinearity. Linearity and homoscedasticity were assessed by examining residual-versus-fitted plots, while the approximate normality of residuals was evaluated using Q–Q plots. Potential non-linear effects of continuous predictors were examined by inspection of scatterplots and residual diagnostics. Sensitivity analyses were performed by replacing the total ERS score with its individual domains (emotional sensitivity, emotional intensity, and emotional persistence). TBSA was modeled primarily as a continuous variable; in sensitivity analyses, TBSA was additionally examined as a categorical variable using a threshold of 30% TBSA (<30% vs. ≥30%). The estimates from these alternative specifications were compared with those from the primary models to assess the robustness of the findings.

All variables included in the primary analyses had complete data for the 256 eligible participants; therefore, no observations were excluded because of missing data, and no imputation was required. All statistical tests were two-sided, with *p* < 0.05 considered statistically significant.

### 2.7. Ethical Considerations

The study was conducted in accordance with the principles of the Declaration of Helsinki. The study protocol was reviewed and approved by the Ethics Committee of the Vietnam National Burn Hospital (Approval No. 285, September 2024). Written informed consent was obtained from all participants before enrollment. The study was reported in accordance with the STROBE recommendations, and the completed STROBE checklist is provided in Supplementary Table S1.

## 3. Results

### 3.1. Participant Characteristics

A total of 256 adult patients with burn injuries met the eligibility criteria and were included in the final analytical cohort. The demographic and clinical characteristics of the study population are summarized in Table 1.

The cohort had a mean age of 39.8 ± 13.2 years, and 211 patients (82.4%) were male. The median TBSA was 18.0% (IQR, 10.0–32.0%). Regarding burn depth, 124 patients (48.4%) had superficial or superficial partial-thickness burns, whereas 132 patients (51.6%) had deep partial-thickness or full-thickness burns.

The mean total ERS score was 40.1 ± 12.6. For emotion-regulation strategies, the mean scores for cognitive reappraisal and expressive suppression were 15.2 ± 4.2 and 12.8 ± 4.1, respectively.

For HRQoL, the mean scores of the three BSHS-B domains included in the present analysis were 68.4 ± 18.7 for Function, 65.7 ± 20.1 for Affect and Relations, and 61.9 ± 22.4 for Skin Involvement. The mean EQ-5D-5L index value was 0.68 ± 0.24.

### 3.2. Association of Burn Severity with Emotional Reactivity

Emotional reactivity was positively correlated with TBSA (r = 0.30, *p* < 0.001). In contrast, burn depth was not significantly associated with emotional reactivity (r_pβ_= −0.08, *p* = 0.18). Both emotion-regulation strategies were significantly correlated with emotional reactivity. Cognitive reappraisal showed a positive correlation with ERS (r = 0.28, *p* < 0.001), while expressive suppression was also positively correlated with ERS (r = 0.25, *p* < 0.001). Patients with TBSA ≥ 30% had significantly higher ERS scores than those with TBSA < 30% (44.8 ± 11.9 vs. 38.2 ± 12.4, *p* < 0.001). ERS scores did not differ significantly according to burn depth. The corresponding association estimates and *p* values are presented in Table 2.

### 3.3. Associations of Psychosocial Characteristics with Health-Related Quality of Life

Higher ERS scores were significantly associated with lower scores across the three BSHS-B domains included in the present analysis. ERS was negatively correlated with Function (r = −0.34, *p* < 0.001), Affect and Relations (r = −0.39, *p* < 0.001), and Skin Involvement (r = −0.28, *p* < 0.001). Cognitive reappraisal was positively correlated with Function (r = 0.21, *p* = 0.001) and Affect and Relations (r = 0.24, *p* < 0.001) but was not significantly correlated with Skin Involvement (r = 0.11, *p* = 0.08). Expressive suppression was negatively correlated with Function (r = −0.18, *p* = 0.004), Affect and Relations (r = −0.30, *p* < 0.001), and Skin Involvement (r = −0.17, *p* = 0.007).

For the generic HRQoL measure, the EQ-5D-5L index was negatively correlated with ERS (r = −0.32, *p* < 0.001) and expressive suppression (r = −0.21, *p* = 0.001), whereas cognitive reappraisal was positively correlated with the EQ-5D-5L index (r = 0.19, *p* = 0.002). The corresponding correlation coefficients and *p* values are presented in Table 3.

### 3.4. Hierarchical Regression Analysis

Complete data was available for all variables included in the primary regression analyses. Accordingly, all hierarchical regression models were based on the full analytical sample of 256 participants, with no observations excluded because of missing data. The analysis focused on the BSHS-B Affect and Relations domain because of its direct conceptual relevance to the psychosocial constructs examined in this study. Table 4 presents the results of the hierarchical multivariable regression analysis. Model 1, adjusted for age and sex, had an adjusted R^2^ of 0.021. Addition of TBSA and burn depth in Model 2 increased the adjusted R^2^ to 0.168 (Δ adjusted R^2^ = 0.147, *p* < 0.001). Further inclusion of emotional reactivity, cognitive reappraisal, and expressive suppression in Model 3 increased the adjusted R^2^ to 0.291 (Δ adjusted R^2^ = 0.123, *p* < 0.001).

In Model 3, higher emotional reactivity was associated with lower BSHS-B Affect and Relations scores (β = −0.31, 95% CI: −0.42 to −0.19, *p* < 0.001). Cognitive reappraisal was positively associated with the outcome (β = 0.17, 95% CI: 0.05 to 0.28, *p* = 0.006), whereas expressive suppression was negatively associated with the outcome (β = −0.14, 95% CI: −0.26 to −0.02, *p* = 0.021). TBSA remained negatively associated with BSHS-B Affect and Relations scores (β = −0.18, 95% CI: −0.31 to −0.06, *p* = 0.004), whereas burn depth was not statistically significant (β = −0.05, 95% CI: −0.16 to 0.06, *p* = 0.34). The standardized regression coefficients and their 95% CIs for Model 3 are presented in Figure 2.

### 3.5. Model Diagnostics and Sensitivity Analyses

No major violations of the assumptions of linearity, homoscedasticity, or normality of residuals were identified. No substantial multicollinearity was identified among the variables included in the models, with VIF values indicating no meaningful multicollinearity.

In sensitivity analyses, replacing the total ERS score with its individual domains-emotional sensitivity, emotional intensity, and emotional persistence—yielded estimates with directions of association comparable to those observed in the primary models. When TBSA was modeled categorically (<30% vs. ≥30%) rather than continuously, the direction and overall pattern of the associations between psychosocial variables and HRQoL remained comparable to those observed in the primary models.

## 4. Discussion

This study suggests that psychosocial characteristics provide additional information for understanding HRQoL beyond conventional measures of burn severity. Greater TBSA was associated with higher emotional reactivity, whereas burn depth was not significantly associated with ERS. Emotional reactivity was consistently associated with poorer burn-specific and generic HRQoL, while cognitive reappraisal showed more favorable and expressive suppression less favorable associations. Importantly, adding these psychosocial characteristics was associated with a 12.3-percentage-point increase in adjusted R^2^ for BSHS-B Affect and Relations, from 0.168 to 0.291. These findings support a biopsychosocial model in which anatomical injury severity and psychological functioning represent complementary dimensions of burn recovery [1,2].

The moderate correlation between TBSA and emotional reactivity (r = 0.30, *p* < 0.001) indicates that greater burn extent is associated with greater emotional reactivity, while also suggesting that injury extent alone does not fully account for individual differences in emotional responses. This is consistent with the systematic review by Spronk et al., in which burn severity was associated with HRQoL in 13 of 18 studies, while psychological and social factors also contributed to outcomes [2]. Prospective studies have similarly shown that HRQoL recovery is influenced by both injury characteristics and individual factors [12,13]. The lack of a statistically significant association between burn depth and ERS suggests that different indicators of burn severity may not show uniform relationships with psychosocial responses in this cohort; this finding should not be interpreted as indicating that burn depth is clinically unimportant.

Emotional reactivity showed the most consistent relationship with HRQoL. Its inverse associations with BSHS-B Function (r = −0.34), Affect and Relations (r = −0.39), Skin Involvement (r = −0.28), and EQ-5D-5L (r = −0.32), all *p* < 0.001, indicate that greater emotional reactivity is associated with poorer perceived health across multiple domains. The strongest association with Affect and Relations is clinically relevant because psychosocial adjustment after burns encompasses emotional well-being, interpersonal functioning, body image, and social participation [1,3]. Emotional reactivity is a dimensional construct rather than a psychiatric diagnosis [4] and may therefore capture psychosocial vulnerability that is not fully represented by conventional diagnostic categories. Long-term studies have likewise demonstrated persistent relationships between psychological characteristics and quality of life after severe burns [14].

The different associations observed for cognitive reappraisal and expressive suppression provide additional evidence that emotion regulation may be relevant to recovery. Cognitive reappraisal was positively correlated with Function, Affect and Relations, and the EQ-5D-5L index, but not with Skin Involvement. Expressive suppression was negatively correlated with all three BSHS-B domains and the EQ-5D-5L index. The stronger inverse association between suppression and Affect and Relations (r = −0.30) suggests that emotion-regulation patterns may be particularly relevant to affective and interpersonal adjustment. Although burn-specific evidence remains limited, broader evidence demonstrates differential relationships between emotion-regulation strategies and mental health [15], while burn research has linked coping styles to quality of life [16]. These findings should remain interpreted as associative, but they suggest that assessment of emotion regulation may complement conventional screening for psychological distress.

The hierarchical analysis provides an important additional contribution. After adjustment for age and sex, TBSA and burn depth increased adjusted R^2^ from 0.021 to 0.168, whereas the addition of ERS, cognitive reappraisal, and expressive suppression increased adjusted R^2^ to 0.291. Adding psychosocial characteristics was associated with a 12.3-percentage-point increase in adjusted R^2^ for BSHS-B Affect and Relations. This finding extends previous evidence that clinical severity and psychological response provide complementary information about HRQoL [2]. It may also help account for heterogeneity in psychosocial outcomes among patients with burn injuries.

The observed associations may also be considered in the broader context of body image and social participation, which have been identified as relevant to psychosocial adjustment after burns. Burn-related scarring and altered appearance can influence self-perception and interpersonal functioning, although the relationship between injury severity and body image is heterogeneous [17,18]. Body-image dissatisfaction has nevertheless been identified as a potential pathway between burn severity and longer-term psychological adjustment [17]. Community participation and peer support are also relevant to recovery, with burn size associated with participation outcomes [19] and peer-support attendance associated with better social interaction [20]. These findings provide contextual support for the strong association between emotional reactivity and the Affect and Relations domain observed in the present study.

Psychosocial consequences of burns extend beyond the domains assessed in the present study and may include body image concerns, social participation, sexuality, sleep, and occupational reintegration [21,22,23,24,25]. These domains have been linked to longer-term psychosocial adjustment and HRQoL after burn injury. Together, the available evidence emphasizes that HRQoL after burn injury is multidimensional and cannot be adequately characterized by wound healing or anatomical severity alone.

The clinical implication is that TBSA and burn depth should remain fundamental measures of injury severity but should not be regarded as sufficient proxies for psychosocial burden. Brief assessment of emotional reactivity and emotion-regulation strategies may provide complementary information for identifying patients who require additional psychological or rehabilitative support. This approach is consistent with contemporary multidisciplinary burn rehabilitation [26], including emerging interventions such as transitional tele-rehabilitation [27] and psychological interventions integrated into rehabilitation. Evidence from low- and middle-income countries further highlights the need for accessible rehabilitation addressing the substantial long-term HRQoL burden after burns [6].

Several limitations should be considered. First, the cross-sectional observational design precludes establishing temporal direction or causal relationships between psychosocial characteristics and HRQoL. Second, the single-center setting and moderate sample size may limit the generalizability of the findings to other burn populations and healthcare settings. Several potentially relevant clinical and anatomical factors, including time since burn injury, total length of hospital stay, number of operative procedures, residual deformities, pain, pruritus, facial involvement, and major functional limb impairment, were not systematically incorporated into the present models. These factors may influence emotional reactivity, emotion-regulation strategies, and HRQoL and may therefore contribute to residual confounding. Future prospective studies should incorporate more comprehensive and prospectively standardized clinical and anatomical variables. Third, the study relied partly on self-reported psychosocial and HRQoL measures, which may be influenced by reporting and response biases. Fourth, several potentially relevant psychosocial factors, including symptoms of depression, anxiety, post-traumatic stress, social support, body image, and perceived stigma, were not included in the primary analytical models and may account for residual confounding. Finally, the absence of longitudinal follow-up precludes determining whether changes in emotional reactivity or emotion-regulation strategies preceding subsequent changes in HRQoL. Prospective longitudinal studies are therefore needed to clarify temporal relationships and evaluate whether these psychosocial characteristics represent potentially modifiable targets for intervention.

Overall, these findings indicate that burn-related HRQoL is associated with both the extent of physical injury and psychosocial functioning. Although TBSA was associated with emotional reactivity, the relatively modest association and the absence of a significant relationship with burn depth suggest that anatomical severity alone does not fully capture patients’ psychosocial burden. The consistent associations between emotional reactivity, emotion-regulation strategies, and HRQoL, together with the increase in adjusted R^2^ after adding psychosocial variables in the hierarchical analysis, support a more comprehensive assessment of recovery that extends beyond conventional measures of burn severity. In clinical practice, this perspective may help guide individualized rehabilitation by recognizing emotional and interpersonal needs alongside physical recovery. However, the cross-sectional observational design does not permit causal conclusions, and prospective longitudinal studies are needed to determine whether changes in psychosocial functioning precede improvements in HRQoL and whether targeted interventions can influence these trajectories.

## 5. Conclusions

Psychosocial functioning represents an important dimension of HRQoL after burn injury and complements conventional measures of physical injury severity. Emotional reactivity and emotion-regulation patterns were associated with patient-reported HRQoL, supporting their consideration in multidimensional assessment and rehabilitation planning. Prospective longitudinal studies are needed to establish the temporal direction of these relationships and determine whether these psychosocial characteristics represent potentially modifiable targets for intervention.

## Figures and Tables

**Figure 1:**
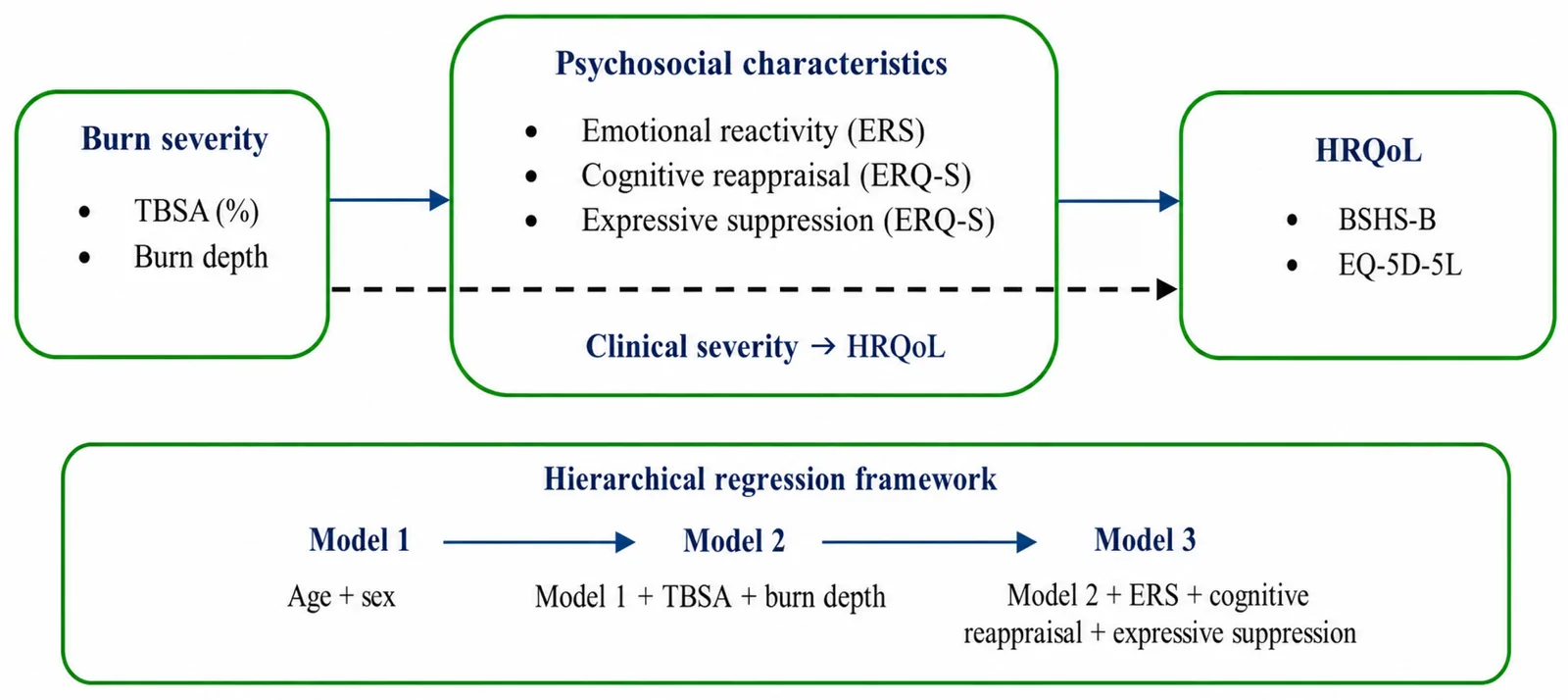
Conceptual and analytical framework of the study, informed by biopsychosocial and health-related quality-of-life frameworks [7,8]. Solid arrows represent prespecified associations examined in the study. The dashed pathway denotes the incremental explanatory contribution of psychosocial variables beyond conventional clinical indicators of burn severity assessed using hierarchical regression. Abbreviations: TBSA, total body surface area; ERS, Emotion Reactivity Scale; ERQ-S, Emotion Regulation Questionnaire–Short Form; BSHS-B, Burn-Specific Health Scale–Brief; HRQoL, health-related quality of life; EQ-5D-5L, EuroQol five-dimensional five-level questionnaire.

**Figure 2:**
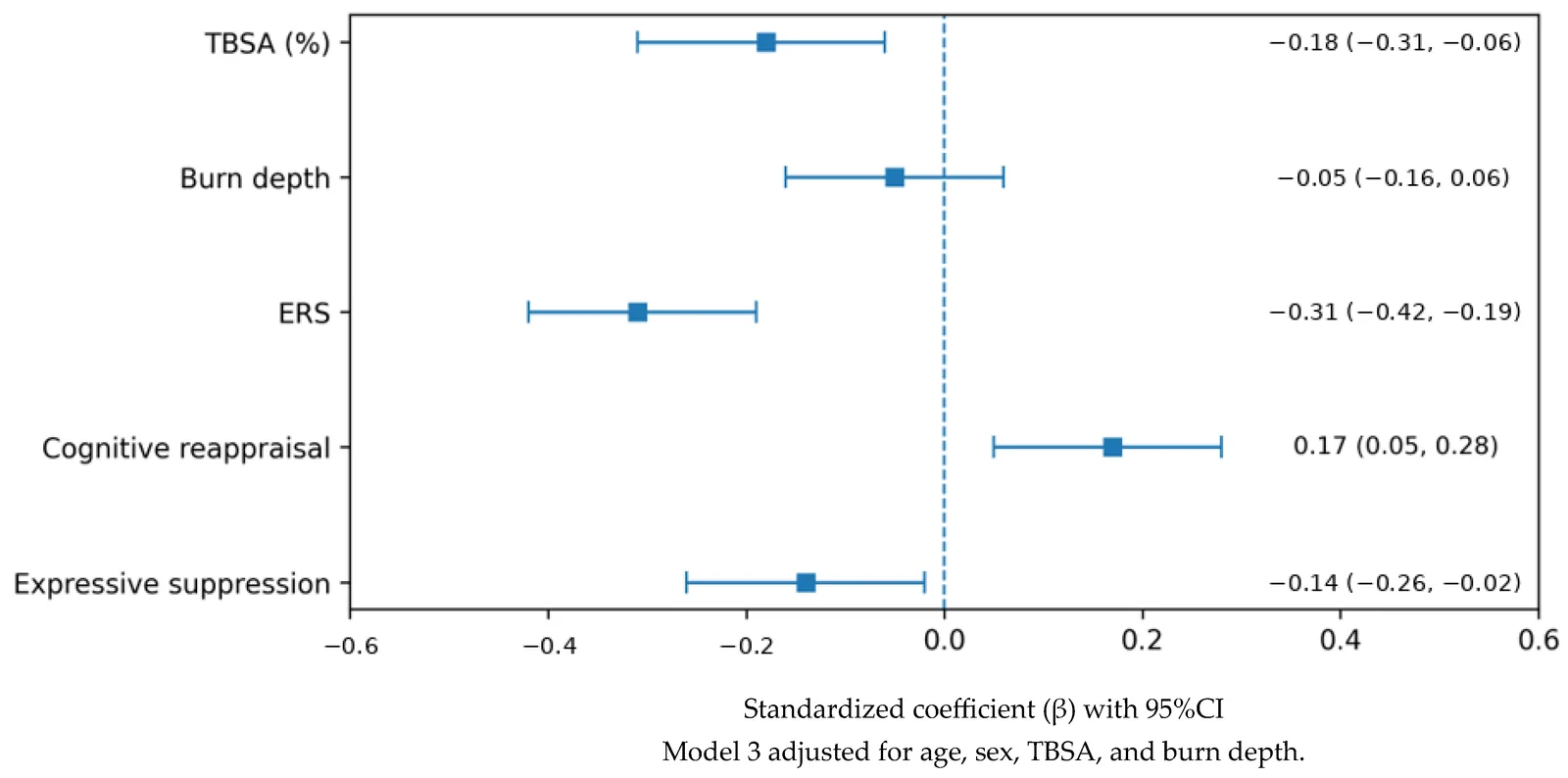
Forest plot of standardized coefficients (β) from Model 3 (hierarchical regression for BSHS-B Affect and Relations).

**Table 1:** Demographic, clinical, psychosocial, and HRQoL characteristics of the study population (*n* = 256).

Variable	Value
**Demographic characteristics**	
Age, years	39.8 ± 13.2
Male sex, *n* (%)	211 (82.4)
**Clinical characteristics**	
TBSA, %, median (IQR)	18.0 (10.0–32.0)
Superficial/superficial partial-thickness burn, *n* (%)	124 (48.4)
Deep partial/full-thickness burn, *n* (%)	132 (51.6)
**Psychosocial characteristics**	
ERS total score	40.1 ± 12.6
Cognitive reappraisal	15.2 ± 4.2
Expressive suppression	12.8 ± 4.1
**Burn-specific HRQoL**	
BSHS-B Function	68.4 ± 18.7
BSHS-B Affect and Relations	65.7 ± 20.1
BSHS-B Skin Involvement	61.9 ± 22.4
**Generic HRQoL**	
EQ-5D-5L index	0.68 ± 0.24

Values are presented as mean ± SD unless otherwise indicated. Abbreviations: TBSA, total body surface area; IQR, Interquartile Range; ERS, Emotion Reactivity Scale; HRQoL, health-related quality of life; BSHS-B, Burn-Specific Health Scale–Brief; EQ-5D-5L, EuroQol five-dimensional five-level questionnaire.

**Table 2:** Associations of clinical and psychosocial variables with emotional reactivity.

Variable	Association with ERS	*p*-Value
TBSA	r = 0.30	<0.001
Burn depth	r_pβ_ = −0.08	0.18
Cognitive reappraisal	r = 0.28	<0.001
Expressive suppression	r = 0.25	<0.001

Note: r, Pearson’s correlation coefficient; r_pβ_, point-biserial correlation coefficient. *p* < 0.05 was considered statistically significant.

**Table 3:** Associations between psychosocial characteristics and HRQoL.

Psychosocial Variable	BSHS-B Function	BSHS-B Affect and Relations	BSHS-B Skin Involvement	EQ-5D-5L Index
ERS	−0.34 **	−0.39 **	−0.28 **	−0.32 **
Cognitive reappraisal	0.21 *	0.24 **	0.11	0.19 *
Expressive suppression	−0.18 *	−0.30 **	−0.17 *	−0.21 *

Note: Values are Pearson’s correlation coefficients (r). *p* < 0.05 was considered statistically significant. * *p* < 0.01; ** *p* < 0.001.

**Table 4:** Hierarchical multivariable regression analysis for BSHS-B Affect and Relations (*n* = 256).

Variable	Model 1 β	Model 2 β	Model 3 β	95% CI	*p*-Value
Age	−0.12	−0.10	−0.08	−0.20 to 0.05	0.21
Male sex	0.06	0.05	0.04	−0.06 to 0.14	0.43
TBSA	—	−0.24 ***	−0.18 **	−0.31 to −0.06	0.004
Burn depth	—	−0.07	−0.05	−0.16 to 0.06	0.34
ERS	—	—	−0.31 ***	−0.42 to −0.19	<0.001
Cognitive reappraisal	—	—	0.17 **	0.05 to 0.28	0.006
Expressive suppression	—	—	−0.14 *	−0.26 to −0.02	0.021
Adjusted R^2^	0.021	0.168	0.291	—	—
Δ adjusted R^2^	—	0.147	0.123	—	—

Note: β, standardized regression coefficient; CI, confidence interval. Model 1 included age and sex; Model 2 additionally included TBSA and burn depth; Model 3 additionally included ERS, cognitive reappraisal, and expressive suppression. *p* < 0.05 was considered statistically significant. * *p* < 0.05; ** *p* < 0.01; *** *p* < 0.001.

## Data Availability

The datasets generated and analyzed during the current study are available from the corresponding author upon reasonable request. The data are not publicly available because they contain potentially identifiable patient information and are subject to institutional ethical restrictions.

## Supplementary Materials

The following supporting information can be downloaded at: https://www.mdpi.com/article/10.3390/ebj7040051/s1, Table S1: STROBE Checklist for Cross-Sectional Studies.

## References

[1] Attoe C., Pounds-Cornish E. (2015). Psychosocial adjustment following burns: An integrative literature review. Burns.

[2] Spronk I., Legemate C.M., Dokter J., van Loey N.E.E., van Baar M.E., Polinder S. (2018). Predictors of health-related quality of life after burn injuries: A systematic review. Crit. Care.

[3] Wisely J.A., Hoyle E., Tarrier N., Edwards J. (2007). Where to start? Attempting to meet the psychological needs of burned patients. Burns.

[4] Nock M.K., Wedig M.M., Holmberg E.B., Hooley J.M. (2008). The Emotion Reactivity Scale: Development, evaluation, and relation to self-injurious thoughts and behaviors. Behav. Ther..

[5] Preece D.A., Petrova K., Mehta A., Gross J.J. (2023). The Emotion Regulation Questionnaire-Short Form (ERQ-S): A 6-item measure of cognitive reappraisal and expressive suppression. J. Affect. Disord..

[6] Mustehsan M.A., Bodla M.A., Shehzad M.M., Abid A., Afzal S. (2026). Health-related quality of life in burn survivors from low- and middle-income countries: A systematic review and targeted comparative analysis of rehabilitation interventions. Burns.

[7] Wiechman S., Hoyt M.A., Patterson D.R. (2020). Using a Biopsychosocial Model to Understand Long-Term Outcomes in Persons with Burn Injuries. Arch. Phys. Med. Rehabil..

[8] Wilson I.B., Cleary P.D. (1995). Linking Clinical Variables with Health-Related Quality of Life: A Conceptual Model of Patient Outcomes. JAMA.

[9] Kildal M., Andersson G., Fugl-Meyer A.R., Lannerstam K., Gerdin B. (2001). Development of a Brief Version of the Burn Specific Health Scale (BSHS-B). J. Trauma.

[10] Herdman M., Gudex C., Lloyd A., Janssen M.F., Kind P., Parkin D., Bonsel G., Badia X. (2011). Development and Preliminary Testing of the New Five-Level Version of EQ-5D (EQ-5D-5L). Qual. Life Res..

[11] Mai V.Q., Sun S., Minh H.V., Luo N., Giang K.B., Lindholm L., Sahlen K.G. (2020). An EQ-5D-5L Value Set for Vietnam. Qual. Life Res..

[12] Van Loey N.E., van Beeck E.F., Faber B.W., van de Schoot R., Bremer M. (2012). Health-related quality of life after burns: A prospective multicenter cohort study with 18 months follow-up. J. Trauma Acute Care Surg..

[13] Wasiak J., Lee S.J., Paul E., Mahar P., Pfitzer B., Spinks A., Cleland H., Gabbe B. (2014). Predictors of health status and health-related quality of life 12 months after severe burn. Burns.

[14] Renneberg B., Ripper S., Schulze J., Seehausen A., Weiler M., Wind G., Hartmann B., Germann G., Liedl A. (2014). Quality of life and predictors of long-term outcome after severe burn injury. J. Behav. Med..

[15] Hu T., Zhang D., Wang J., Mistry R., Ran G., Wang X. (2014). Relation between emotion regulation and mental health: A meta-analysis review. Psychol. Rep..

[16] Rothman D.J., Sutter M., Perrin P.B., LiBrandi H., Feldman M.J. (2016). Coping styles and quality of life in adults with burn. Burns.

[17] Huang Y.K., Su Y.J. (2021). Burn severity and long-term psychosocial adjustment after burn injury: The mediating role of body image dissatisfaction. Burns.

[18] Cleary M., Kornhaber R., Kaji Thapa D., West S., Visentin D. (2020). A quantitative systematic review assessing the impact of burn injuries on body image. Body Image.

[19] Ryan C.M., Shapiro G.D., Rencken C.A., Griggs C.L., Jeng J.C., Hickerson W.L., Marino M., Goverman J., Kazis L.E., Schneider J.C. (2022). The impact of burn size on community participation: A Life Impact Burn Recovery Evaluation (LIBRE) study. Ann. Surg..

[20] Grieve B., Shapiro G.D., Wibbenmeyer L., Acton A., Lee A., Marino M., Jette A., Schneider J.C., Kazis L.E., Ryan C.M. (2020). Long-term social reintegration outcomes for burn survivors with and without peer support attendance: A Life Impact Burn Recovery Evaluation (LIBRE) study. Arch. Phys. Med. Rehabil..

[21] Connell K.M., Coates R., Wood F.M. (2013). Sexuality following burn injuries: A preliminary study. J. Burn Care Res..

[22] Lerman S.F., Owens M.A., Liu T., Puthumana J., Hultman C.S., Caffrey J.A., Smith M.T. (2022). Sleep after burn injuries: A systematic review and meta-analysis. Sleep Med. Rev..

[23] Mason S.T., Esselman P., Fraser R., Schomer K., Truitt A., Johnson K. (2012). Return to work after burn injury: A systematic review. J. Burn Care Res..

[24] Watson J.D., Perrin P.B. (2022). Relations among stigma, quality of life, resilience, and life satisfaction in individuals with burn injuries. Eur. Burn J..

[25] Al Hanna R., Cofré Lizama L.E., Amatya B., Galea M.P., Khan F. (2024). Rehabilitation in adults with burn injury: An overview of systematic reviews. Disabil. Rehabil..

[26] Bayuo J., Wong F.K.Y., Chung L.Y.F. (2024). Effect of a transitional tele-rehabilitation programme on quality of life of adult burn survivors: A randomised controlled trial. Clin. Rehabil..

[27] Ruan J., Huo T., Zhang Y., Ruan Q., Xi M., Ru T., Liu S. (2025). Effect of Acceptance and Commitment Therapy on psychological wellbeing and physical function in rehabilitation patients after burns: A randomized controlled trial. Burns.

