# Supplementary material for: Emotional Reactivity and Emotion Regulation in Relation to Health-Related Quality of Life After Burn Injury: A Cross-Sectional Observational Study
Source: Eur Burn J. 2026 Sep 29;7(4):51. doi: 10.3390/ebj7040051 (PMC13692327; doi:10.3390/ebj7040051)
Supplement: Supplementary file 1 [file ebj-07-00051-s001.zip › ebj-4521628-supplementary.pdf]

SUPPLEMENTARY TABLE S1. STROBE CHECKLIST FOR CROSS-SECTIONAL STUDIES

Manuscript: "Emotional Reactivity and Emotion Regulation in Relation to Health-Related Quality of Life After Burn Injury: A Cross-Sectional Observational Study

Section/Topic	Item No.	Checklist item	Report on Section / Location
**TITLE AND ABSTRACT**			
Title and abstract	**1a**	Indicate the study's design with a commonly used term in the title or the abstract	Title; Abstract (p. 1)
	**1b**	Provide in the abstract an informative and balanced summary of what was done and what was found	Abstract (p. 1)
**INTRODUCTION**			
Background/rationale	**2**	Explain the scientific background and rationale for the investigation being reported	1. Introduction (pp. 2–3)
Objectives	**3**	State specific objectives, including any prespecified hypotheses	1. Introduction (pp. 2–3)
**METHODS**			
Study design	**4**	Present key elements of study design early in the manuscript	2.1. Study Design (p. 3)
Setting	**5**	Describe the setting, locations, and relevant dates, including periods of recruitment, exposure, follow-up, and data collection	2.1. Study Design; 2.2. Data Collection; 2.3. Participants (pp. 3–4)
Participants	**6a**	Give the eligibility criteria, and the sources and methods of selection of participants	2.3. Participants (p. 4)
	**6b**	For matched studies, matching criteria and number of exposed and unexposed	N/A — not a matched study
	**6c**	For cohort studies, report the methods of follow-up	N/A — cross-sectional study; no follow-up
Variables	**7**	Clearly define all outcomes, exposures, predictors, potential confounders, and effect modifiers. Give diagnostic criteria, if applicable	2.3. Participants; 2.4. Clinical and Psychosocial Assessment; 2.5. Health-Related Quality of Life; 2.6. Statistical Analysis (pp. 4–6)
Data sources/measurement	**8**	For each variable of interest, give sources of data and details of methods of assessment (measurement). Describe comparability of assessment methods if there is more than one group	2.2. Data Collection; 2.4. Clinical and Psychosocial Assessment; 2.5. Health-Related Quality of Life (pp. 4–5)
Bias	**9**	Describe any efforts to address potential sources of bias	2.2. Data Collection; 2.6. Statistical Analysis; 4. Discussion (pp. 4–6, 9–12)
Study size	**10**	Explain how the study size was arrived at	2.3. Participants (p. 4)
Quantitative variables	**11**	Explain how quantitative variables were handled in the analyses. If applicable, describe which groupings were chosen and why	2.6. Statistical Analysis (pp. 5–6)
Statistical methods	**12a**	Describe all statistical methods, including those used to control for confounding	2.6. Statistical Analysis (pp. 5–6)
	**12b**	Describe any methods used to examine subgroups and interactions	N/A — no subgroup or interaction analyses were performed
	**12c**	Explain how missing data were addressed	2.6. Statistical Analysis (p. 6)
	**12d**	If applicable, describe analytical methods taking account of sampling strategy	N/A — census-based inclusion; no complex sampling design

Section/Topic	Item No.	Checklist item	Report on Section / Location
	**12e**	Describe any sensitivity analyses	2.6. Statistical Analysis; 3.5. Model Diagnostics and Sensitivity Analyses (pp. 5–6, 9)
	**12f**	Describe any methods used to address loss to follow-up	N/A — cross-sectional study; no follow-up
**RESULTS**			
Participants	**13a**	Report numbers of individuals at each stage of study—e.g., numbers potentially eligible, examined for eligibility, confirmed eligible, included in the study, completing follow-up, and analysed	2.3. Participants; 3.1. Participant Characteristics (pp. 4, 6). Final analytical cohort: 256; earlier screening-stage counts are not reported.
	**13b**	Give reasons for non-participation at each stage	2.3. Participants (p. 4). Eligibility/exclusion criteria are reported; counts and reasons for non-participation at each stage are not reported.
	**13c**	Consider use of a flow diagram	Not provided; no participant flow diagram was included.
Descriptive data	**14a**	Give characteristics of study participants and information on exposures and potential confounders	3.1. Participant Characteristics; Table 1 (pp. 6–7)
	**14b**	Indicate number of participants with missing data for each variable of interest	2.6. Statistical Analysis; 3.4. Hierarchical Regression Analysis (p. 6, p. 8). Complete data were available for all primary-analysis variables; no missing observations were excluded.
	**14c**	Summarise follow-up time (e.g., average and total amount)	N/A — cross-sectional study; no longitudinal follow-up
Outcome data	**15**	Report numbers of outcome events or summary measures over time	3.2–3.4; Tables 2–4 (pp. 7–9). Summary measures are reported; no longitudinal follow-up was conducted.
Main results	**16a**	Give unadjusted estimates and, if applicable, confounder-adjusted estimates and their precision (e.g., 95% CI). Make clear which confounders were adjusted for and why they were included	3.2–3.4; Tables 2–4 (pp. 7–9)
	**16b**	Report category boundaries when continuous variables were categorized	2.6. Statistical Analysis; 3.2. Association of Burn Severity with Emotional Reactivity (pp. 5–7). TBSA sensitivity analysis used <30% vs. ≥30%.
Other analyses	**17**	Report other analyses done.g., analyses of subgroups and interactions, and sensitivity analyses	3.5. Model Diagnostics and Sensitivity Analyses (p. 9)
**DISCUSSION**			
Key results	**18**	Summarise key results with reference to study objectives	4. Discussion; 5. Conclusions (pp. 9–12)
Limitations	**19**	Discuss limitations of the study, taking into account sources of potential bias or imprecision	4. Discussion (pp. 11–12)
		Discuss both direction and magnitude of any potential bias	4. Discussion (pp. 11–12)
Interpretation	**20**	Give a cautious overall interpretation of results considering objectives, limitations, multiplicity of analyses, results from similar studies, and other relevant evidence	4. Discussion; 5. Conclusions (pp. 9–12)
Generalisability	**21**	Discuss the generalisability (external validity) of the study results	4. Discussion; 5. Conclusions (pp. 11–12)
**OTHER INFORMATION**			
Funding	**22**	Give the source of funding and the role of the funders for the present study and, if applicable, for	Funding statement (p. 12)

Section/Topic	Item No.	Checklist item	Report on Section / Location
		the original study on which the present article is based	
